# Frequency and Clinical Significance of Elevated IgG4 in Rheumatoid Arthritis: A Systematic Review

**DOI:** 10.3390/biomedicines10030558

**Published:** 2022-02-26

**Authors:** Rajalingham Sakthiswary, Syahrul Sazliyana Shaharir, Asrul Abdul Wahab

**Affiliations:** 1 Department of Medicine, Universiti Kebangsaan Malaysia Medical Centre, Kuala Lumpur 56000, Malaysia; sazliyana@hotmail.com; 2 Department of Immunology, Universiti Kebangsaan Malaysia Medical Centre, Kuala Lumpur 56000, Malaysia; saw@ppukm.ukm.edu.my

**Keywords:** arthritis, rheumatoid, immunoglobulin G, immune system

## Abstract

Immunoglobulin (Ig)G4 is a unique protein molecule and its role in autoimmune diseases remains elusive and controversial. Accumulating evidence suggests a pathogenic role of IgG4 in rheumatoid arthritis (RA). Rheumatoid factors (RF) in RA can recognize the Fc domains of IgG4 to form RF-IgG4 immune complexes that may activate the complement system leading to synovial injury. The aim of this article was to systematically review the literature from the past 2 decades to determine the frequency of elevated IgG4 and its clinical significance in RA. We comprehensively searched the Pubmed, Scopus, and Web of Science databases with the following terms: “IgG4”, “rheumatoid arthritis”, and “immunoglobulin G4”, and scrutinized all of the relevant publications. Based on the selection criteria, 12 studies were incorporated, which involved a total of 1715 RA patients. Out of 328 subjects from three studies, the pooled frequency of elevated non-specific IgG4 was 35.98%. There was a significant positive correlation between the IgG4 levels and the RA disease activity based on DAS-28 measurements (r = 0.245–0.253) and inflammatory markers, i.e., erythrocyte sedimentation rate (ESR) and C-reactive protein (CRP) levels (r = 0.262–0.389). Longitudinal studies that measured the serial levels of IgG4 consistently showed a decline in the concentrations (up to 48% less than baseline) with disease modifying anti-rheumatic drug (DMARD) treatment. Current evidence suggests that serum IgG4 levels are significantly elevated in RA compared to the general population. This review indicates that IgG4 is a promising biomarker of disease activity and tends to decline in response to DMARD therapies. Biologic therapies have revolutionized the therapeutic armamentarium of RA in the recent decade, and IgG4 appears to be a potential treatment target.

## 1. Introduction

Immunoglobulin (Ig)G accounts for 80% of the total immunoglobulins in human serum, and can be divided into four subclasses, i.e., IgG1 (60–70%), IgG2 (15–20%), IgG3 (5–10%), and IgG4 (4–6%). Each of these has different immunological properties and functions [1]. Immunoglobulins play a pivotal role in autoimmune diseases such as rheumatoid arthritis (RA), systemic lupus erythematosus (SLE), and myasthenia gravis. RA is a chronic inflammatory joint disease with a complex pathogenesis. The orchestrated interaction of a wide array of cytokines, autoreactive B cells, and T cells underpin the mechanisms in RA. The sera of RA patients tend to typically exhibit a wide variety of autoantibodies [2]. Rheumatoid factors (RF), which are the predominant autoantibodies in RA, have Fab segments, which react with the Fc portion of the IgG molecule to generate IgM (RF)-IgG immune complexes, which can stimulate the complement system and trigger a cascade of events in the synovial microenvironment [3]. IgG4 molecules have stirred much interest among researchers in the past decade, ever since IgG4-related disease (IgG4-RD) was endorsed in 2011 [4]. The striking difference between IgG4-RD and RA is the presence and frequency of joint involvement. IgG4-RD is a multisystemic disorder, with arthritis being reported in only 10% of patients [5]. The common forms of presentations of IgG4-RD include autoimmune pancreatitis, sclerosing cholangitis, sclerosing sialedenitis, orbital disease, and retroperitoneal fibrosis [6]. RA, on the other hand, is primarily a disease of the joints. The presence of arthritis is mandatory to diagnose RA [7]. 

IgG4 is a poorly understood molecule with controversial roles in the immune system. Traditionally, IgG4 has been viewed as a “non-inflammatory” molecule, which dampens rather than incites immune activation. This is due to the unique molecular structure of IgG4, whereby the heavy chains in each IgG4 molecule have inefficient disulphide bridges due to a single amino acid difference in the hinge region [8]. In hemi-IgG4 molecules, one heavy chain may covalently bind with one light chain, and then dissociate from each other and re-associate randomly with other hemi-IgG4 molecules. This phenomenon is known as the “Fab arm exchange”, which exclusively occurs in the IgG4 subclass [9]. This half-antibody exchange generates antibodies that are capable of binding two different antigens, but are rarely able to form large immune complexes [10]. Based on these theories, IgG4 have a limited ability to form immune responses.

Nevertheless, accumulating evidence suggests a pathogenic role of IgG4 based on its correlations with disease activity and the severity of certain disease entities [4,11]. IgG4 may theoretically bind to Fc receptors on macrophages and eosinophils, and facilitate the presentation of extracellular antigens to CD4+ T lymphocytes [12,13]. Some recent publications have implicated IgG4 autoantibodies in the pathogenesis of RA. Histopathological findings of IgG4 infiltration in the rheumatoid synovium and elevated serum levels of IgG4 in RA patients lend further credence to the above notion [3,14,15].

As far as we know, there are no published systematic reviews focusing on IgG4 in RA. Hence, the purpose of this systematic review was to gather and scrutinize all available literature in the past few decades to determine the pooled frequency of elevated IgG4 and its clinical significance. 

## 2. Materials and Methods

### 2.1. Search Strategy

**Figure 1:**
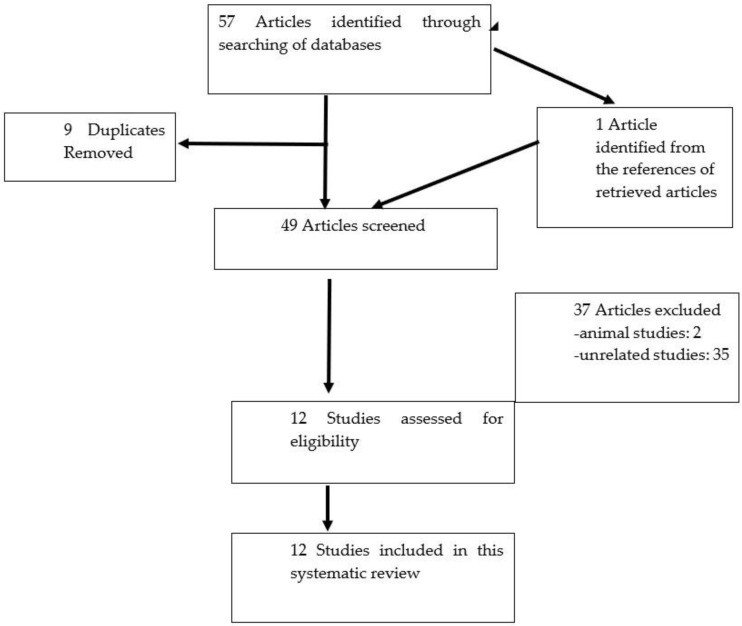
The algorithm for the selection of studies in this systematic review.

We comprehensively searched the Pubmed, Scopus, and Web of Science databases with the following terms: “IgG4”, “immunoglobulin G4”, and “rheumatoid arthritis”, and tracked all of the publications. All three authors independently performed a literature search by title and abstract screening using the Endnote software. In the event of uncertainty, the full text of the article was obtained and assessed. Disagreements were resolved by a consensus-based discussion. Only articles that were approved after much scrutiny by all were finally included in the review. To minimize the selection, information, and confounding biases, the PICOT (patient/population, intervention, control, outcome, time) approach was employed to develop the inclusion and exclusion criteria [16]. The population in this review referred to patients with RA, intervention in most studies included treatment with disease modifying anti-rheumatic drugs, and the outcome was the serum IgG4 levels. A clear search protocol reduced the ambiguity in the selection process of the articles. In order to achieve extensive coverage without missing any relevant articles, the references of all retrieved articles were reviewed. This systematic review was conducted in accordance with the standards set by the Preferred Reporting Item for Systematic Review and Meta-Analysis (PRISMA) Statement [17]. Figure 1 summarizes our search strategy. 

### 2.2. Inclusion Criteria

All adult human studies written in English that looked into IgG4 in RA were included. Conference abstracts with sufficient data were considered eligible. 

### 2.3. Exclusion Criteria

We excluded studies published before 2000. Furthermore, articles in other languages, case reports, case series, animal studies, editorials, and review articles were excluded.

### 2.4. Data Extraction

After compiling the relevant studies, the authors extracted the relevant data from each paper, including year of publication, country, study design, study population, frequency of subjects with elevated IgG4, mean/median IgG4 levels in RA, and the correlations with clinical and biochemical markers. The Newcastle−Ottawa Scale [18] (Table 1) was used for the quality assessment of the 11 included observational studies. The above scale is not applicable for randomized trials. Scores of ≥3 were considered as low risk of bias, whereas <3 were judged as high risk. Disagreements among the authors were solved through discussions and a consensus was reached. 

## 3. Results

### 3.1. Study Characteristics Proteomic Analysis of IgG4

Based on the selection criteria, 12 studies were incorporated, which involved a total of 1715 RA patients. Among the included twelve research works, four were from Asia [14,15,19,20], seven from Europe [21,22,23,24,25,26,27], and one from North America [28]. All of the studies in this series were observational, except for one randomized trial [23]. There were seven cross-sectional studies [14,15,19,20,22,24,27] and five longitudinal studies [21,23,25,26,28] included in this review. The quality assessment of the observational studies based on the Newcastle−Ottawa scale revealed that six articles were of low-risk bias (≥3 points) and the remaining five were of high-risk bias (<3 points).

IgG4 levels were detected using three methods, i.e., ELISA in seven studies [21,22,23,24,26,27,28], immunonephelometry in four studies [14,15,19,20], and radioimmunoassay in a single study [25]. Of note, all Asian studies used the immunonephelometry method of testing IgG4. The studies that performed the immunonephelometric quantification of IgG4 stored the samples between −80 to −70 degrees Celsius after the samples were processed in a centrifugal separator. The total levels of IgG and IgG4 were determined with liquid reagent kits [15,19]. The levels of IgG4 specific-anti-citrulinated cyclic peptide (CCP) antibodies were determined using the ELISA kit containing a CCP-coated plate with horseperoxidase-conjugated anti-human IgG4 antibodies [28].

### 3.2. Frequency of Elevated IgG4 in Rheumatoid Arthritis

There were five studies that analyzed the non-specific IgG4 levels in RA [14,15,19,20,23] (Table 2). There were two studies that did not provide data on the frequency of subjects with raised levels of IgG4 [20,23]. Out of 328 subjects from three studies, the pooled frequency of elevated IgG4 was 35.98%. The studies used different kits with variable units of measurements, i.e., g/L, mg/L and mg/dL. Calculation of effect size was not performed as there were only two studies [15,19] that provided the mean values of IgG4. 

There were four studies that investigated the levels of IgG4 specific to citrullinated cyclic peptide (CCP) [21,22,27,28] and two studies on citrullinated cyclic fibrinogen (CCF) [24,26] (Table 3). The pooled frequency of elevated IgG4 anti-CCP was 330 out of 581 subjects (56.79%).

### 3.3. Clinical Significance of IgG4 in Rheumatoid Arthritis

#### 3.3.1. IgG4 and Disease Activity

There were four studies that investigated the association of serum IgG4 levels with the RA disease activity [14,15,19,20]. Of note, two of the studies were from the same group of researchers [14,15]. Kim et al. [19] found significant correlations between serum IgG4 levels and DAS28-ESR (r = 0.245; *p* = 0.016), and with ESR (r = 0.262; *p* = 0.010). In keeping with these findings, Chen et al. [15] revealed that IgG4 levels correlated positively with CRP (r = 0.373), ESR (r = 0.389), and DAS28 (r = 0.253; all *p* < 0.05) [4]. The Pearson correlation coefficient r value from these studies for the correlation between IgG4 levels and the RA disease activity based on DAS-28 measurements ranged from 0.245–0.253, whereas for inflammatory markers, i.e., ESR and CRP levels, it was 0.262–0.389. The r values that fell between 0.2–0.4, in general, reflected a weak to moderate strength in the relationships of the aforementioned variables [29]. There was a trend towards higher IgG4 levels in the high disease activity group compared to the moderate, low, and remission groups, although statistical significance was not achieved. 

In one of the studies, the synovial samples of RA patients had a median IgG4 positive(+) plasma cells count of 83 (10–192)/mm^2^ and a median ratio of IgG4+/IgG+ plasma cells of 19.1 (8.4–31.5). Both of them were positively correlated with ESR, CRP, and serum IgG4 (r = 0.216–0.394, all *p* < 0.05) [14]. 

#### 3.3.2. IgG4 and Treatment Response 

There were four longitudinal studies [21,23,26,28] that evaluated the changes in the levels of IgG4 with therapy. The therapies used included biologic disease modifying anti-rheumatic drugs (DMARDs) such as tocilizumab [28], adalimumab [26], conventional DMARDs [21], and an experimental agent that was oral bovine type II collagen [23]. All of these studies except for one [23] consistently showed a decline in the IgG4 levels with treatment. There was a parallel decrease in the disease activity of the subjects. Bos et al. [26] disclosed that although all types of IgG (IgG1–4) decreased with treatment, the good responders based on European League Against Rheumatism (EULAR) response criteria [30] had the greatest decline in antibody levels, and this effect was most pronounced for IgG4 (48% reduction). Similarly, Carbone et al. [28] found a 2–3-fold reduction in IgG4 levels with tocilizumab therapy, but not in IgG1 levels, despite IgG1 being the most frequent IgG subtype. 

Among the subjects who were treated with adalimumab, secondary failure to this biologic therapy was due to the formation of anti-drug antibody, which was IgG4 in up to 29% of the subjects [25].

## 4. Discussion

To the best of our knowledge, this is the first systematic review in the literature on IgG4 in RA. Our pooled analyses showed that IgG4 levels were significantly elevated in RA patients (35.98%) compared to the frequencies reported in healthy individuals. Various studies have found that the frequency of elevated IgG4 in healthy subjects ranged from 0–2.5% [19,31]. In keeping with our findings, several studies have reported higher frequencies of elevated IgG4 in autoimmune diseases such as Sjogren syndrome, systemic lupus erythematosus, myasthenia gravis, and eosinophilic granulomatous polyangiitis [32,33]. A serum IgG4 concentration of above 135 mg/dL has been widely accepted as the cut-off value to define “elevated IgG4” and as a criterion for the diagnosis of IgG4-related disease [34]. 

There was a significant positive correlation between the IgG4 levels and RA disease activity based on the findings of all three studies that performed correlation analyses between the above-mentioned parameters. Of note, all three studies used the same composite clinical disease activity tool, i.e., DAS28-ESR, which may partially explain the similarity in the findings. Disease activity in RA reflects synovial inflammation, which is due to the effects of circulating cytokines, such as interleukin (IL)-1, IL-6, and tumor necrosis factor (TNF) α. The synthesis of IgG4 in vitro was regulated by IL-6. IL-6 may enhance IgG4 production through IL-21 expressed in CD4+ T cells [35], which in turn promotes the differentiation of B cells into antibody-secreting plasma cells [36]. The link between IL-6 and IgG4 may explain the relationship between the latter and RA disease activity. The pro-inflammatory nature of IL-6 is well established in RA and it plays important roles in the regulation of the immune response, inflammation, and bone metabolism [37]. The reported association in the studies need not necessarily imply causation of RA disease activity directly by IgG4. Nevertheless, elevated IgG4 levels may indicate a relapse of RA. The conventional biomarkers of disease activity widely used by clinicians in day-to-day clinical practice are ESR and CRP. Clinicians may consider IgG4 as an adjunct biomarker in this regard, but not for diagnostic purposes. 

The independent role of IgG4 in RA remains elusive, although there is some supporting evidence based on the histopathological analysis by Chen et al. [14,15]. The studies demonstrated marked infiltration of RA synovium by IgG4-positive plasma cells, which were correlated with a total synovitis score, inflammatory infiltration subscore, CD3-positive T cells, CD20-positive B cells, and CD38-positive plasma cells. This finding suggested that IgG4 was potentially a culprit molecule in RA rather than an innocent bystander. It is tempting to speculate that the fibrosis observed in the RA synovium could be secondary to the upregulation of a fibrogenic cytokine, i.e., transforming growth factor (TGF)-β by IgG4 [38]. This postulation is based on our knowledge on IgG4-RD and its striking histological feature, which is fibrosis [39].

**Figure 2:**
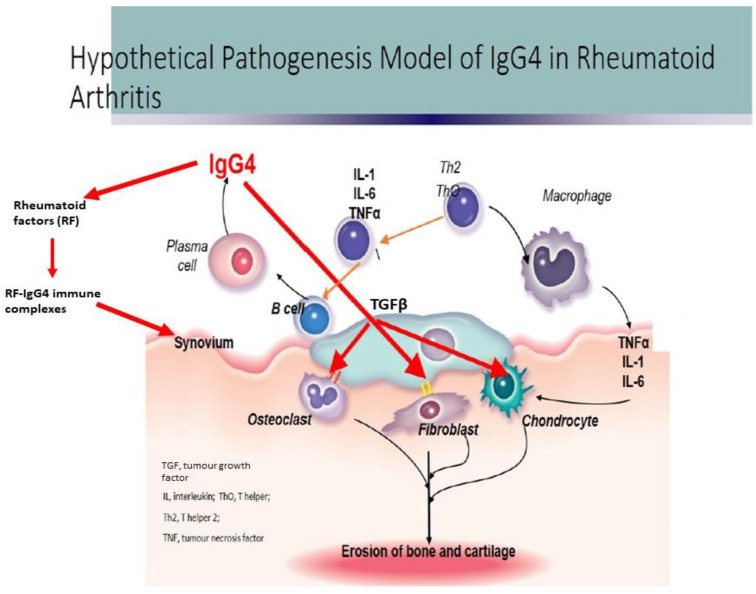
Hypothetical pathogenesis model on the role of IgG4 in rheumatoid arthritis.

Rheumatoid factors (RF) in RA can recognize the Fc domains of IgG4 to form RF-IgG4 immune complexes that may activate the complement system, leading to synovial injury [40]. Although IgG1 is the most frequent isotype against citrullinated cyclic peptide, Bos et al. [26] proposed that prolonged exposure to autoantigens might lead to changes in the IgG4/IgG1 antibody ratio switching to an IgG4-dominated response. Figure 2 illustrates the theoretical role of IgG4 in the pathogenesis of RA. 

The evidence from this systematic review suggests that IgG4 is a reliable biomarker of treatment response. The results from the studies in this regard were consistent. There are a few hypothetical explanations for the above. DMARD therapy tends to inhibit IgG4 production via TNFα inhibition [41]. Furthermore, IgG4 levels, unlike IgG1 levels tend to decline with therapy among responders due to disruptions in the chronic stimulation by citrullinated proteins [42]. Citrullination is inflammation-dependent and is hence suppressed by DMARD therapies, which have anti-inflammatory properties. IgG1 levels are stable and inflammation-independent as they are predominantly produced by long-lived plasma cells, whereas IgG4 levels are produced by short-lived plasma cells, which are driven by citrullinated proteins [43].

We acknowledge the limitations of this systematic review. Most of the studies were conducted in Europe and Asia, which may limit the representativeness of the results to a certain extent. There are racial and ethnic disparities with regard to the disease characteristics and clinical outcomes in RA [44]. We were unable to calculate the effect size for the correlation between the RA disease activity and IgG4, as well as the difference in the means of IgG4 across the various categories of RA patients due to the lack of relevant numerical data in the included studies. From the limited data from a few studies, conclusions cannot be made firmly and may appear speculative. Moreover, several articles with ambiguous data description were excluded, which may affect the pooled frequency. 

## 5. Conclusions

Current evidence suggests that the serum IgG4 levels are elevated in RA compared to the general population. This review indicates that IgG4 is a promising biomarker of disease activity, and tends to decline in response to DMARD therapies. Thus, IgG4 could serve as an alternative modality in RA to assess patients’ disease severity. There are several theories with regard to the pro-inflammatory role of IgG4. Further research is necessary to substantiate these hypotheses.

## Figures and Tables

**Table 1:** The Newcastle−Ottawa Scale for quality assessment of observational studies.

Studies	Selection				Comparability	Outcome			Total Score
	Exposed Truly Representative of Average	Selection of Non Exposed from the Same Community	Exposure Ascertained by Secure Record or Interview	Demonstration of Outcome of Interest Not Present at the Start of the Study	Study Controls for Other Variables	Follow Up Long Enough for Outcome to Occur	Complete Follow Up for All Subjects Accounted for	Subjects Lost to Follow Up Unlikely to Introduce Bias	
Chen et al. (2014)	✓	✓	✓	x	x	x	x	x	3
Lin et al. (2010)	✓	x	✓	x	x	x	x	x	2
Kim et al. (2020)	✓	x	✓	x	x	x	x	x	2
Chen et al. (2021)	✓	✓	✓	x	x	x	x	x	3
K. Martinsson et al. (2016)	✓	x	x	x	x	x	x	x	1
Carbone et al. (2013)	✓	x	✓	x	x	✓	✓	X	4
Engelmann et al. (2008)	✓	x	x	x	x	x	x	x	1
Bos et al. (2009)	✓	x	✓	x	x	✓	✓	x	4
Engelmann et al. (2018)	✓	x	✓	x	x	x	✓	x	3
Chapuy-Regaud et al. (2005)	✓	x	x	x	x	x	x	x	1
van Schouwenburg et al. (2012)	✓	x	✓	x	x	✓	x	x	3

**Table 2:** Summary of rheumatoid arthritis studies on non-specific IgG4.

Year	Authors	Country	Study Design & Study Population	Frequency of RA Patients with Raised IgG4	Mean/Median IgG4 Levels in RA	IgG4 Detection Method	Key Findings
2014	Chen et al. [15]	China	Cross sectional 136 RA patients	46%	1.52 ± 1.27 g/L	Immunonephelometry	51% patients had elevated IgG4/IgG ratio. The mean IgG4 of the untreated patients was 1.82 ± 1.39 g/L, which was significantly higher than that of the treated patients (1.39 ± 1.20 g/L; *p* = 0.044) IgG4 of high disease activity group was significantly higher than that of the remission group (*p* = 0.003). CRP and ESR, of the elevated IgG4 group were significantly higher than those of the normal IgG4 group (CRP: 38 ± 42 mg/L vs. 25 ± 33 mg/L; ESR: 70 ± 42 mm/h vs. 48 ± 32 mm/h; all *p* < 0.05). IgG4 level correlated positively with CRP (r = 0.426) and ESR (r = 0.315; both *p* < 0.05). RF and anti-CCP Ab levels of the elevated IgG4 group were significantly higher than those of the normal IgG4 group (RF: 513 ± 636 IU/mL vs. 245 ± 392 IU/mL; anti-CCP Ab: 256 ± 243 U/mL vs. 162 ± 199 U/mL; both *p* < 0.05), IgG4 correlated positively and significantly with synovial IgG4-positive plasma cells but no significant correlation of IgG4 with total synovitis score or subscores.
2021	Chen et al. [14]	China	Cross sectional 96 active RA patients	49 (51.0%)	1.38 (0.86–2.42) g/L (median)	Immunonephelometry.	RA patients with elevated IgG4 had significantly higher levels of ESR [90 (64–116) mm/h vs. 61 (38–75) mm/h], CRP [46.20 (17.20–74.20) mg/L vs. 18.90 (9.46–49.20) mg/L], DAS28-ESR [6.3 (5.6–7.4) vs. 5.7 (4.7–6.4)], SDAI [34.2 (25.3–48.8) vs. 27.8 (18.9–35.9)] all *p* < 0.05]. They also showed significantly higher synovial counts of IgG4+ plasma cells [106 (39–249) /mm^2^ vs. 68 (3–123) /mm^2^], and higher ratio of IgG4+/IgG+ plasma cells [26.3 (15.5–38.0)% vs. 15.2 (0.9–24.7)%, all *p* < 0.05]. There were 10 (10.4%) patients showing elevated serum IgG4 and IgG4-related synovitis based on synovial biopsy.
2010	Lin et al. [20]	China	Cross sectional 72 RA patients		365.5 (72.85–1377.5) mg/L (median)	Immunonephelometry.	When the patients were divided according to the clinical activity, the IgG subclass concentrations were similar between the two groups (*p* > 0.05).
2020	Kim et al. [19]	Korea	Cross sectional 128 participants (RA: 96; healthy controls: 17; Osteoarthritis: 11; and IgG4-related disease: 4)	6.3%	48.0 ± 45.4 mg/dL (mean)	Immunonephelometry.	The mean serum IgG4/IgG ratio in patients with RA was 3.5 ± 2.8% (range 0.2–16.9%). None of the healthy controls or patients with osteoarthritis had elevated serum IgG4 levels. A significant correlation was found between serum IgG4 levels and the Disease Activity Score-28 with erythrocyte sedimentation rate (r = 0.245; *p* = 0.016).
2010	Farboud et al. [23]	United Kingdom	Longitudinal (24 weeks) 55 RA patients			ELISA	For IgG1, IgG2, IgG3 and IgG4 subclasses, in the 0.5-mg of oral bovine type II collagen group in which the best clinical response was observed, there was statistically significant decreases observed in the IgG2 and IgG3 subclasses (*p* = 0.047, *p* = 0.046) but not in IgG4.

RA: rheumatoid arthritis, ESR: erythrocyte sedimentation rate, CRP: C-reactive protein, RF: rheumatoid factor, CCP: citrulinated cyclic peptide.

**Table 3:** Summary of Rheumatoid Arthritis studies on specific types of IgG4.

Year	Authors	Country	Study Design & Study Population	Frequency of RA Patients with Raised IgG4	Type of IgG4	IgG4 Detection Method	Key Findings
2016	K. Martinsson et al. [27]	Sweden	Cross sectional 504 with recent onset RA (untreated)	59%	IgG4 anti-CCP	ELISA	Among those who were RF positive, 79% subjects tested positive for IgG4. IgG anti-CCP subclasses that associate with HLA-DRB1/SE are IgG1 and IgG4. Increased proportion of IgG4 anti-CCP-positive cases that were not associated with smoking. The fractions of IgG4 anti-CCP did not differ significantly between ever and never-smokers.
2013	Carbone et al. [28]	USA	Longitudinal 8 patients with active RA were treated with tocilizumab (TCZ) monotherapy or in combination with non-biologic DMARDs over 6 months; serum samples were obtained at (0 month), 1 month, 3 months, and 6 months		IgG4 anti-CCP	ELISA	Over the 6 months of treatment, there was a prominent four-fold reduction in the levels of IgG4 (*p* = 0.06). The levels of IgG4 were markedly decreased in all but one patient. Pronounced reduction (2–3 fold) in the serum levels of IgG4-specific anti-CCP Abs in all patients (*p* = 0.011), but no statistically significant reduction in the levels of IgG1-anti-CCP Abs (*p* = 0.185).
2014	Engelmann et al. [22]	Germany	Cross sectional 77 RA patients	33 (42.86%) patients with anti-CCP antibodies are positive for the IgG4 subclass	IgG4 anti-CCP	ELISA	Even though IgG1 is the predominant subclass among antibodies against CCP and anti-citrullinated vimentin (MCV) in RA, IgG4 was conspicuously elevated. Elevated IgG4 titers among auto-antibodies in RA are indicative of a Th2-biased environment.
2018	Engelmann et al. [21]	Germany	Longitudinal 34 ACPA-positive RA were monitored for 3 months after therapy		IgG4 anti-CCP	ELISA	3 months after therapy, the responders showed a significant decrease in IgG4 ACPA levels, and this was independent of the individual treatment regimen.
2009	Bos et al. [26]	Netherlands	Longitudinal 180 patients treated with adalimumab for 28 weeks		IgG4 ACF	ELISA	The median reduction in anti-citrullinated fibrinogen (ACF) levels was 31% for total IgG, 29% for IgG1, 40% for IgG4, and 22% for the IgG4/IgG1 ACF ratio in the infliximab cohort. In adalimumab-treated patients, ACF levels declined 14% for total IgG and IgG1, and 36% for IgG4 ACF; the IgG4: IgG1 ratio was reduced by 24%. European League Against Rheumatism good responders had the greatest decline in antibody levels, and this effect was most pronounced for IgG4 (48% reduction). The IgG4/IgG1 ACF ratio preferentially decreased in patients with adequate therapeutic adalimumab levels.
2005	Chapuy-Regaud et al. [24]	France	Cross sectional 186 RA	21.3% (30/141) had IgG1-AhFibA in combination with IgG4-AhFibA	IgG4 ACF	ELISA	IgG4-Antihuman fibrinogen (AhFibA) observed much more frequently and at higher titers than IgG3- or IgG2-AhFibA. AhFibA were mainly IgG1 and, to a lesser extent, IgG4.
2012	van Schouwenburg et al. [25]	Netherlands	Longitudinal 271 RA patients monitored for 3 years of adalimumab treatment	29% of the patients had detectable IgG4	IgG4 against adalimumab	Radio immunoassay	The proportion IgG4 of total IgG against adalimumab varied widely between patients. IgG4 was found to contribute significantly to the anti-drug antibody (ADA) response in some patients.

RA: rheumatoid arthritis; DMARD: disease modifying anti-rheumatic drug.

## Data Availability

The data presented in this study are available upon request from the corresponding author.
